# Characterization and Toxic Potency of Airborne Particles Formed upon Waste from Electrical and Electronic Equipment Waste Recycling: A Case Study

**DOI:** 10.1021/acsenvironau.3c00034

**Published:** 2023-11-03

**Authors:** Inger Odnevall, Marianne Brookman-Amissah, Franca Stábile, Mikael T. Ekvall, Gunilla Herting, Marie Bermeo Vargas, Maria E. Messing, Joachim Sturve, Lars-Anders Hansson, Christina Isaxon, Jenny Rissler

**Affiliations:** † Department of Chemistry, Division of Surface and Corrosion Science, KTH Royal Institute of Technology, SE-100 44 Stockholm, Sweden; ‡ AIMES−Center for the Advancement of Integrated Medical and Engineering Sciences at Karolinska Institute and KTH Royal Institute of Technology, SE-100 44 Stockholm, Sweden; § Department of Neuroscience, Karolinska Institute, SE-171 77 Stockholm, Sweden; ∥ Ergonomics and Aerosol Technology, Lund University, SE-221 00 Lund, Sweden; ⊥ Department of Biological and Environmental Sciences, University of Gothenburg, SE-405 30 Gothenburg, Sweden; # Department of Biology, Aquatic Ecology, Lund University, SE-223 62 Lund, Sweden; ∇ Solid State Physics, Lund University, Box 118, 221 00 Lund, Sweden; ○ Bioeconomy and Health, RISE Research Institutes of Sweden, SE-223 70 Lund, Sweden; ◆ NanoLund, Lund University, SE-221 00 Lund, Sweden

**Keywords:** electronic waste, WEEE, aerosols, environmental dispersion, characterization, ecotoxicity

## Abstract

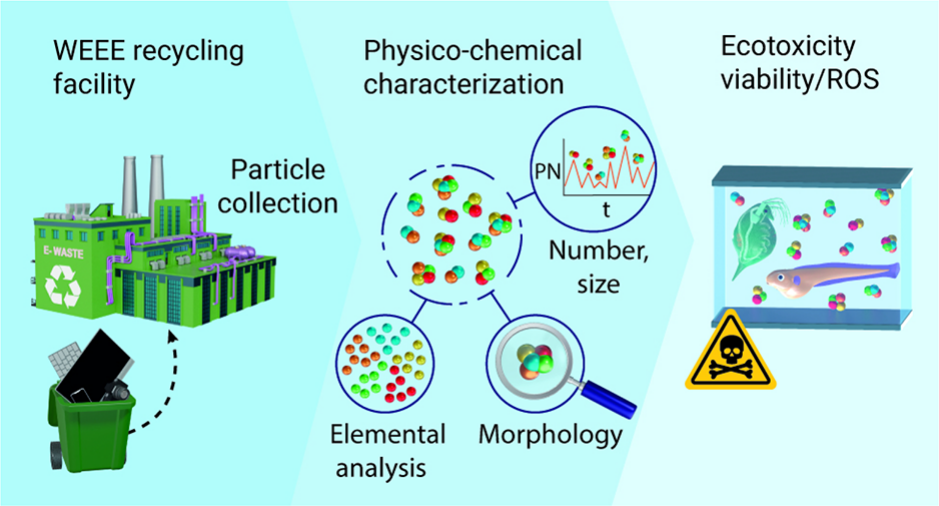

Manual dismantling, shredding, and mechanical grinding of waste from electrical and electronic equipment (WEEE) at recycling facilities inevitably lead to the accidental formation and release of both coarse and fine particle aerosols, primarily into the ambient air. Since diffuse emissions to air of such WEEE particles are not regulated, their dispersion from the recycling plants into the adjacent environment is possible. The aim of this interdisciplinary project was to collect and characterize airborne WEEE particles smaller than 1 μm generated at a Nordic open waste recycling facility from a particle concentration, shape, and bulk and surface composition perspective. Since dispersed airborne particles eventually may reach rivers, lakes, and possibly oceans, the aim was also to assess whether such particles may pose any adverse effects on aquatic organisms. The results show that WEEE particles only exerted a weak tendency toward cytotoxic effects on fish gill cell lines, although the exposure resulted in ROS formation that may induce adverse effects. On the contrary, the WEEE particles were toxic toward the crustacean zooplankter *Daphnia magna*, showing strong effects on survival of the animals in a concentration-dependent way.

## 1 Introduction

Handling of waste from electrical and electronic equipment (WEEE) is in the EU regulation via the waste electrical and electronic equipment (no. 2012/19/EU) and the RoHS (2011/65/EU) directives, the latter to ensure minimum risks to human health and the environment. The WEEE directive mandates that each EU country ensures the annual collection, recycling, and recovery of electrical goods, aiming for a minimum annual rate of more than 4 kg per person. In 2017, this corresponded to 3.7 million tons of WEEE, predominantly originating from large household appliances (51.8%), consumer equipment and photovoltaic panels (14.8%), IT and telecommunication equipment (14.6%), small household appliances (10.2%), and other sources (8.7%).^1^ The industry producing electrical and electronic equipment is one of the fastest growing worldwide, and thus, the WEEE will grow accordingly. In the newly adopted policy by the European parliament, it is stated that a toxic-free environment and circular economy should be fully operational by 2050.

Manual dismantling, shredding, and mechanical grinding of WEEE at recycling facilities inevitably lead to the accidental formation and release of both coarse and fine particles into the ambient air and thus pose a risk for inhalation or skin contact.^2−4^ Adverse effects on human health caused by such particles have been reported for both routes of exposure.^5,6^ Furthermore, as WEEE recycling plants typically are open spaces, the emitted airborne WEEE particles can spread to the adjacent environment. In many countries that are in the process of economic and social growth, WEEE is handled and treated in open air.^7^

The WEEE waste contains a range of metals such as copper (Cu), zinc (Zn), iron (Fe), aluminum (Al), lead (Pb), and gold (Au), which can be recovered and recycled. Metals are typically the most abundant component followed by plastics and glass and may also contain flame retardants and neurotoxins.^8^ The recycling process is complex and involves several steps including manual handling and mechanical and chemical treatments, potentially generating particles of which a significant amount may be nanosized (NPs), emitted to the surrounding air.

A range of measures to mitigate the risks associated with WEEE particles have been proposed including the use of protective equipment during the recycling process, the implementation of effective air filtration systems, and the development of safer recycling technologies. An improved understanding of the physicochemical properties and toxicity of particles emitted during the WEEE treatment is therefore crucial for the development of appropriate regulations and guidelines for safe handling and disposal of electronic waste.

Research on WEEE particles has focused on characterizing size, morphology, composition, and toxicity of the particles generated during the recycling process.^9,10^ However, studies on the emissions from European treatment plans for WEEE are still scarce,^2−4^ and studies of potential ecotoxicological effects of the emitted particles are even fewer. The results of the few studies performed show that the composition of WEEE particles varies depending on the type of electronic device, its age, and the processing methods used for its disposal. Moreover, particles inhaled and absorbed into the human body may lead to adverse health effects such as respiratory and cardiovascular diseases. For example, effects on human health and the environment close to recycling plants have recently been reported.^11^

Since diffuse emissions of WEEE particle aerosols are not regulated, many treatment processes are performed in buildings/under roof with low, or no, barriers to the ambient air. Thus, there is an evident risk for their environmental dispersion into the atmosphere and that they eventually end up in aquatic systems via rain and surface runoff from land. Such environmental dispersion of WEEE particles are reported in the literature.^3,12−14^ This may lead to an accumulation over time where waterbodies act as a natural sink for the released WEEE particles in nature. The nonintentional dispersion of airborne WEEE particles carried by the wind and their atmospheric deposition into the terrestrial and aquatic ecosystems may result in adverse ecotoxicological effects on aquatic organisms.^12^

Overall, relevant case studies including the characterization of submicrometer particles provide valuable insight into potential risks associated with airborne emissions of WEEE particles on humans and the environment.

The aim of this interdisciplinary paper, combining expertise in aerosol physics, material science, surface chemistry, biology, and ecotoxicology, was to collect and characterize WEEE particle aerosols generated at a Nordic waste recycling facility from a particle concentration, shape, and bulk and surface composition perspective and assess their toxic potency toward aquatic organisms. Ecotoxicity testing was conducted on *Daphnia magna*, a common model species of OECD standard testing toxicity of substances, and using a gill cell line of Rainbow trout, as fish is on top of the food chain.

## 2 Materials and Methods

### 2.1 Waste Recycling Facility and WEEE Treatment Step

Particles emitted to the air were monitored and sampled indoors at a facility where recycling various waste streams, of which one is WEEE (waste of electrical and electronic equipment) took place. The processing of the WEEE covered manual sorting and dismantling (components for reuse are separated and hazardous components removed), mechanical shredding, and crushing followed by various separation steps.^15^ This study focused on particles emitted after the mechanical treatment in an industrial hall where the shredded WEEE first was transported and then further sorted. In-depth measurements of particle aerosol formation and exposure assessments at the same recycling facility (including three waste flows of WEEE, metal scrap, and cables) are reported elsewhere.^4^

### 2.2 Particle Sampling from Air and Online Instrumentation

For the ecotoxicity studies, particles were collected during two consecutive working days using a high-volume cascade impactor (BGI900; Mesa Laboratories, USA BGI Inc., Waltham, MA, USA). With an air flow of 0.9 m^3^/min, particles of an aerodynamic diameter <1 μm (PM1) were collected onto Teflon filters (Whatman, GE Healthcare, diameter 150 mm, TE38). Particles sized >1 μm were removed by using an impactor stage upstream. Sampling was conducted during several 2–3 h periods, exchanging filters and cleaning the impactor stage before each sampling period.

In parallel, the aerosol particles were characterized with respect to number concentrations and mass concentrations using online instrumentation. A condensation particle counter, CPC, was used (model 1720, Brechtel, USA) to measure the number concentration of particles sized >7 nm in diameter with a time resolution of 30 s. A DustTrak (model DRX 8533, TSI Inc. USA) instrument was used for optical online measurements of the mass concentration with a time resolution of 1 min. The instrument was equipped with an impactor stage allowing only particles sized <1 μm (PM1) to pass. Since the technique relies on correct assumptions of refractive index and particle density, it was mainly used to study temporal variations in the PM1 mass concentration.

The mass concentrations of total dust (TD) and respirable dust (RD) were assessed following standard practice for gravimetric analysis. TD corresponds to the total mass of airborne particles, collected with “open face” filters. The RD corresponds to the respirable fraction of particles, i.e., with an aerodynamic diameter less than ∼4 μm. RD was collected using a cyclone as a preseparator, removing the largest particles (BGI4L, BGI Inc., USA; cutoff 4 μm at 2.2 L/min). The filters utilized for the collection of TD and RD were 37 mm mixed cellulose ester filters with a pore size of 0.8 μm, sourced from Millipore.

Additionally, RD for scanning electron microscopy and energy-dispersive spectroscopy (SEM/EDS) and X-ray photoelectron spectroscopy (XPS) analysis was collected on polycarbonate filters (37 mm, SKC Inc., pore size 0.4 μm) and TD for analysis of organic (OC) and elemental carbon (EC) was collected using a quartz filter (25 mm, Pallflex Tissuquartz preheated 2500QAT-UP). The OC and EC were determined using thermal-optical analysis (TOA), performed according to the EUSAAR_2 protocol.^16^ Chemical analyses, described in Section 2.3, were made using the same filters as used for determining mass concentrations by the gravimetric analysis.

All filters were mounted in conductive three-piece filter cassettes (SureSeal, SKC Inc., USA) as described elsewhere.^4^

Particles were also collected for analysis by means of X-ray absorption spectroscopy (XAS) using an impactor, allowing size resolved sampling of particles with diameters between 40 nm and 10 μm. The impactor used was a custom-built multinozzle low pressure impactor, with a flow rate of 10 L/min and downstream pressure of 0.13 bar. The impactor was equipped with 12 impactor stages with size cut-offs (in aerodynamic particle diameter) for the different stages of 0.04, 0.09, 0.15, 0.22, 0.36, 0.58, 0.81, 1.07, 1.68, 2.69, 4.46, and 8.55 μm.

### 2.3 Chemical Properties and Particle Shape

#### 2.3.1 Chemical Composition of Individual Particles and Particle Shape Using Scanning Electron Microscopy with Energy-Dispersive Spectroscopy (SEM/EDS)

The morphology and elemental composition of a large collection of particles collected on polycarbonate filters were analyzed by means of SEM/EDS using a ZEISS Gemini 500 scanning electron microscope equipped with a Multim Max 170 EDS detector. The SEM/EDS analysis was conducted at an accelerating voltage ranging from 10 to 15 kV and a 30 μm aperture. The polycarbonate filter samples were mounted onto a Si wafer and coated with a thin layer (approximately 3 nm) of Pt/Pd (80:20). Following data acquisition, a postanalysis was performed, involving the filtering out of overlapping signals and excluding C, Pt, and Pd, which are associated with the filter and coating materials.

The shape and chemical composition of collected particles for the XPS investigation and of the particles extracted from the filters for the ecotoxicity studies were determined using a PHILIPS FEI XL30 instrument with an Oxford X-Max 20 mm2 SDD EDS detector (Oxford Instruments) using an acceleration voltage of 15 kV.

#### 2.3.2 Elemental Analysis Using Inductively Coupled Plasma Mass Spectrometry (ICP-MS), Graphite-Furnace-Atomic Absorption Spectroscopy (GF-AAS), and Particle-Induced X-ray Emission (PIXE)

Total amounts of aluminum (Al), arsenic (As), Fe, cadmium (Cd), cobalt (Co), chromium (Cr), Cu, manganese (Mn), nickel (Ni), Pb, zinc (Zn), vanadium(V), barium (Ba), tallium (Tl), and gallium (Ga) in the particles were determined by means of ICP-MS (Thermo iCAP Q, Thermo Scientific, Bremen, Germany). Analysis was performed using the kinetic energy discrimination mode with helium as collision gas. The filters were placed in Teflon flasks and dissolved in 1 mL of concentrated nitric acid overnight at 70 °C and then further diluted (1:10, 1:100, and 1:200, when necessary) in 2 vol % nitric acid to a final volume of 5 mL. The detailed description of the method is given elsewhere.^4^

Total Si concentrations were determined using GF-AAS (PerkinElmer PinAAcle900T) on samples adjusted to a pH of >8 to ensure Si to be in solution. With calibration standards of 0, 600, and 1000 μg/L, the limit of detection (LOD) and limit of quantification (LOQ) were determined to be 3 and 10 μg/L, respectively. Quality control samples of known concentrations were analyzed after every sixth sample.

The elemental composition of assemblies of collected particles of different size fractions (see Section 2.2), and of the stock solution prepared for the ecotoxicity investigation, was analyzed by means of PIXE using a focused proton beam of ∼1 cm^2^. The elemental content was estimated by assuming homogeneous coverage of particles at the filter surfaces. More details are given elsewhere.^4,17^

#### 2.3.3 Surface Characterization and Oxidation State by Means of X-ray Photoelectron Spectroscopy (XPS) and X-ray Absorption Near-Edge Spectroscopy (XANES)

Information on the composition and oxidation state of the particles (outermost 5–10 nm) was acquired by means of XPS using a Kratos AXIS Supra instrument using a monochromatic Al Kα source operating at 15 mA and 15 kV. Spectra were charge-corrected to the main line of C 1s (C–C and C–H) set to 284.8 eV. Survey scan analyses were carried out on two separate areas sized 300 × 700 μm using a pass energy of 160 eV and high-resolution spectra using a pass energy of 20 eV.

XANES was used to study the chemical form of a subselection of metals in this case Cu, Zn, Cr, and Fe. These metals were selected since they were in high enough concentrations in the collected particles to retrieve high quality spectra, and their toxic potency toward aquatic organisms is partly governed by their chemical form (and dose). The measurements were performed at the Balder beamline at the 3 GeV ring at MAX IV, Lund, Sweden.^18^ The 3 GeV storage ring was operated at ∼250 mA. Monochromatization was achieved with a pair of Si111 crystals, and the beam was focused to ∼200 × 100 μm. The measurements of the WEEE particles were performed in fluorescence mode using an energy disruptive Ge-detector. The XANES spectra for the K-edge of respective element were scanned (Cu at 8979 eV, Zn at 9659 eV, Cr at 5989 eV, and Fe at 7112 eV). A library of previously generated XANES reference spectra for Cu, Zn, and Cr recorded in transmission mode at the K-edges of the respective element (retrieved at earlier occasions at Balder) was used for comparison. For Fe, only the three most common oxides were used for comparison (Fe, FeO, Fe_2_O_3_, and Fe_3_O_4_) with the acquired XANES data.

The investigated samples included four filters with total dust (TD)—two filters from this study and two filters collected 1 year earlier at the same site—as well as particles collected at the different impactor stages corresponding to particle size fractions of 100–150 nm, 220–360 nm, 1.5–2.7 μm, and 2.7–4.5 μm. Due to the low concentration of particles per surface area, and the fact that the beam spot was only 100 × 100 μm, not all TD samples, including the particle size fraction of 100–150 nm, could be analyzed for all four metals. Particles collected and extracted by means of methanol from the filters for the ecotoxicity testing (see below) were investigated in parallel to assess if the extraction method would change the chemical form of the metals of interest.

The data was preprocessed (summation of spectra, background subtraction, normalization, and interpolation onto a common energy grid) and analyzed further using the ATHENA software package.^19^

### 2.4 Ecotoxicological Studies

#### 2.4.1 WEEE Particle Extraction from Filters and Particle Dispersion Preparation for the Ecotoxic Studies

Particle removal from the collecting filters was conducted following the protocol of Mesa laboratories.^20^ In short, the filters were cut into 2 × 2 cm squares and immersed in analytical grade pure methanol (MeOH) in a 250 mL glass flask and sonicated for 60 min (the sonication bath temperature did not exceed 35 °C). The sonicated solution was then transferred to a clean glass flask. The sonication procedure was repeated with new MeOH. After this final step, the sonicated solution was pipetted into preacid-cleaned 12 mL glass vials and dried in a vacuum evaporator (SpeedVac HT-4X Evaporator; GeneVac Ltd., Ipswich, UK).

**Figure 1:**
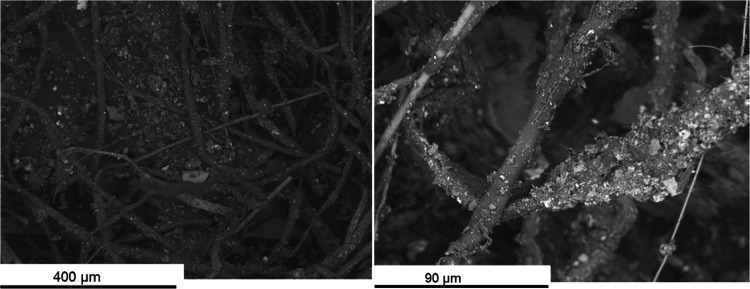
Secondary electron SEM images of particles attached onto fibers originating from the collecting filters after the methanol particle extraction treatment.

After implementing the extraction procedure to detach particles from the collecting filters into individual tubes for further testing as described above, some fibrous material with attached particles was transferred into the vessel, as shown in Figure 1.

The particles with fibers were immersed in 4 mL of artificial freshwater (0.0065 g/L NaHCO_3_, 0.00058 g/L KCl, 0.0294 g/L CaCl_2_·2 H_2_O, and 0.0123 g/L MgSO_4_·7 H_2_O, Sigma-Aldrich, Sweden) to enable as many of these particles to detach from the fibers as possible and enable the preparation of particle dispersions of known concentrations for the ecotoxicity investigation. The pH of each vessel was adjusted to 6.2 using 50% NaOH followed by bath sonication for 1 min. The remaining fibrous matter (floating in solution) was manually removed with a spatula or tweezer leaving only minor amounts of fibrous material. This may result in some unavoidable loss of particles, though the soluble particle fraction remains in solution. The actual PM1 concentration in each vessel was therefore estimated based on PIXE results of the stock solution prepared for the ecotoxicity measurements. This estimation assumed that the mass fraction of each metal in PM1 was preserved during the filter extraction and the fiber detachment. The metals, which had a content that was high enough to be detected in all four replicates of the stock solution using PIXE, were used (i.e., Fe, titanium (Ti), Mn, Cr, Cu, Zn, and Pb, see Section 3.4.1) to calculate the approximate mass of PM1 WEEE particles. These calculations resulted in a final mass of 1.5 mg WEEE particles per glass vessel (in total >50 vessels). These vessels were pooled to achieve the investigated particles doses (2.3–75 mg/L).

#### 2.4.2 Target Organisms for Ecotoxicological Assessments

Previous studies have shown that both crustacean zooplankton^21,22^ and fish^23,24^ are vulnerable to different types of nanosized particles. Therefore, a zooplankton species (*D. magna*) was selected, which constitutes a crucial link in the food chain from primary producers (algae) and higher trophic levels. This species is also commonly used in OECD standard protocols to test chemicals.^25^ Moreover, as fish are generally at the top of the food chain and have been shown to be strongly affected by NPs, both with respect to behavior and metabolism, we also used a gill cell line of Rainbow trout (*Oncorhynchus mykiss*) as an end point for our ecotoxicological assessments on the effects of WEEE particles as they enter aquatic ecosystems.

##### 2.4.2.1 Rainbow Trout Cell Line: Cytotoxicity and Oxidative Stress

Rainbow trout (*O. mykiss*) gill Waterloo 1 (RTgill-W1) cells were cultured according to protocols described elsewhere.^26^ Cells were seeded with L-15 plus 5% fetal bovine serum (FBS) in 96-well plates at a density of 40,000 cells per well and incubated at 19 °C for 24 h in a Memmert incubator.

The WEEE particles were dispersed in different exposure solutions; a Leibovitz-15 cell culture medium (L-15) or L-15/ex saline buffer and phosphate-buffered saline (PBS)^27^ to a concentration of ∼0.4 mg/mL based on the protocol described above and further diluted to obtain desired exposure concentrations ranging from 2.3 to 75 mg/L.

Preseeded 96-well plates were exposed to 100 μL of WEEE particle dispersions in six replicates and incubated for 48 h at 19 °C. Three technical replicates and three cell passages were included to account for variability in the results. Copper sulfate (CuSO_4_) (Sigma-Aldrich) was used as a control.

Post exposure, changes in cell morphology were evaluated using a microscope before the cells were rinsed with L-15/ex solution following measurement of cytotoxicity by AlamarBlue (Invitrogen), 5-carboxyfluorescein diacetate acetoxy methyl ester (CFDA-AM, Thermo Fisher Scientific), and Neutral Red assays performed as described elsewhere.^26^

Fluorescence intensity was measured using a Spectramax Gemini M microplate reader at excitation/emission wavelengths of 532/590, 485/535, and 532/680 nm for AlamarBlue, CFDA-AM, and Neutral Red, respectively. Fluorescence values were normalized against the controls and presented as percentage of cell viability.

The generation of reactive oxygen species (ROS) during exposure and after 48 h of exposure to WEEE was measured by using the 6-carboxy-2′7′-dichlorofluorescein diacetate (DCFH-DA) assay according to methods previously presented elsewhere.^26^ Single-point fluorescence measurements were periodically made over 18 h, following direct WEEE exposure and kinetically measured 3 h following 48 h of exposure to WEEE at excitation/emission wavelengths of 485/535 nm using a Spectramax Gemini M microplate reader. Data was represented as the generation of ROS.

##### 2.4.2.2 *D. magna*: Acute Toxicity Test

The toxicity of the WEEE particles was assessed on the freshwater crustacean zooplankter *D. magna* using a 72 h acute toxicity test. A WEEE particle dispersion was prepared as described above. Two concentrations, 37 and 74 mg L^–1^, of WEEE particles were investigated alongside a control containing tap water only. Juvenile *D. magna* (2–3 days old) were placed in individual 50 mL Falcon tubes containing a total of 40 mL of test media (WEEE particles dispersed in tap water). Each treatment was replicated 10 times, and the survival was registered for 72 h. Statistical differences in survival at the end of the experiment were evaluated using Kaplan–Meier survival analysis in GraphPad Prism 7e for Mac OSX.

## 3 Results and Discussion

### 3.1 Physical Particle Characteristics in Air

**Figure 2:**
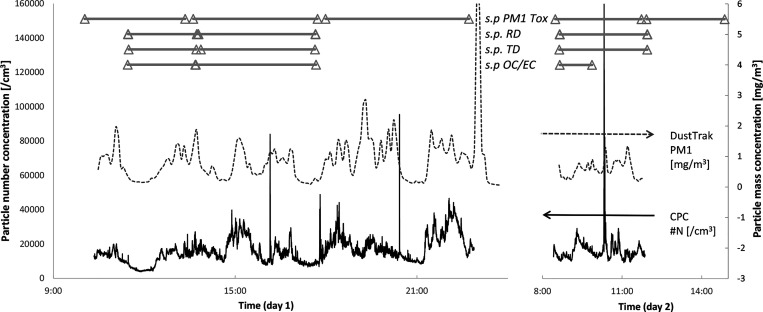
Time-resolved (online) data on particle concentration (from CPC, left axis) and mass concentrations (from DustTrak, right axis). Sampling periods for the filter collection are indicated. “s.p PM1 Tox” represent the sampling periods of PM1 for the toxicity studies, “s.p. RD” and “s.p. TD” represent the sampling periods of respirable dust and total dust, respectively, and “s.p. OC/EC” represents the sampling periods of filters for OC/EC analysis.

The measured time series of airborne particle concentrations during two consecutive days originating from the WEEE treatment are presented in Figure 2 in terms of particle mass (PM1) and particle number (PN). Time periods for the collection of particles for the ecotoxicity studies along with the filters for gravimetrical and chemical analyses are indicated in the figure. Averaged results on total dust (TD), respirable dust (RD), particle number concentration (PN), and concentrations of total elemental carbon (EC) and total organic carbon (OC) are presented in Table 1.

**Table 1:** Summary of the Physical Particle Characteristics per Volume Air Including TD (total dust), RD (respirable dust), PN (Particle Number Concentration), (T)EC (Total Elemental Carbon), and (T)OC (Total Organic Carbon)

	**concentration**	**relative standard deviation [%]**
TD [mg/m^3^]	3.3 ± 1.9	58
RD [mg/m^3^]	0.27 ± 0.05	18
PN [cm^3^]	15,633 ± 6,786	43
(T)EC [μg/m^3^]	23 ± 6	26
(T)OC [μg/m^3^]	435 ± 31	7

**Figure 3:**
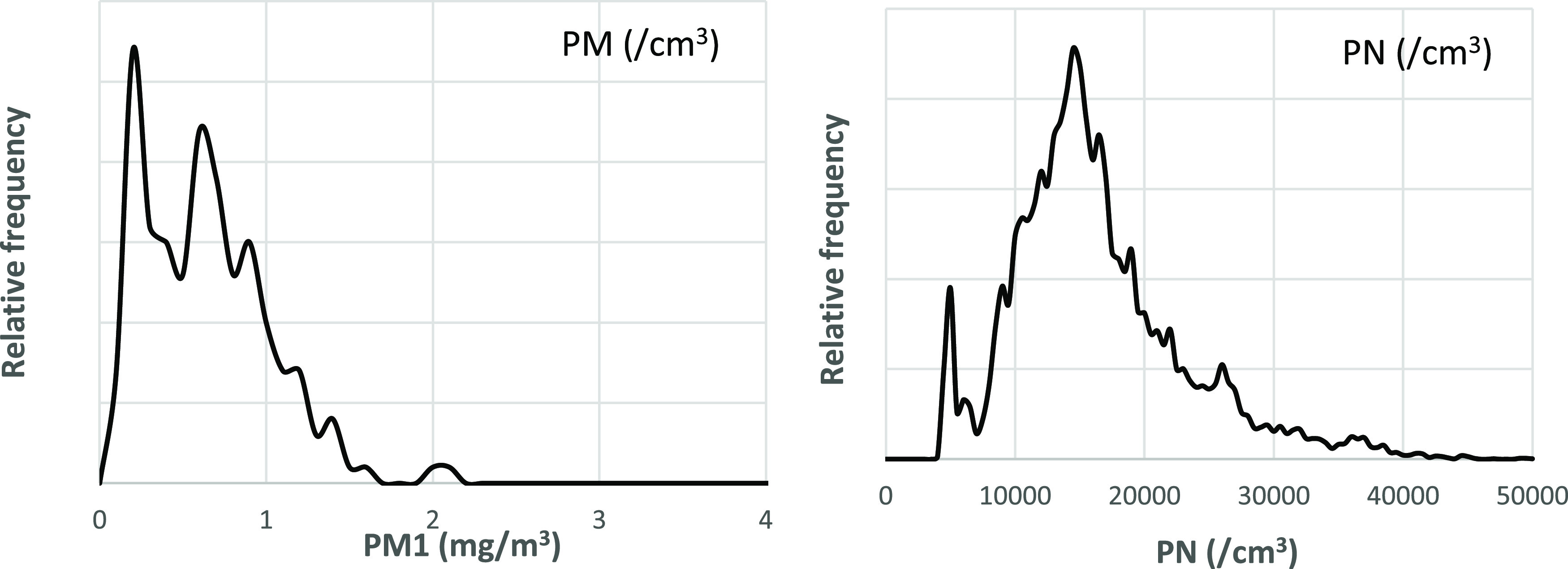
Frequency distribution plots from the online instruments measuring PM1 (mg/m^3^), PN (/cm^3^), and BC (μg/m^3^) during working hours (07:00–23:00).

The results from the online measurements are also summarized in density plots (Figure 3) and the measured mass concentration given (Table 1) together with the results from the thermal-optical analysis (TOA) and average PN from the online instruments. The TD levels were ∼3 to 4 mg/m^3^, while the RD was, as expected, considerably lower (0.27 mg/m^3^). PN concentrations were ∼15,000 particles/cm^3^. According to the TOA analysis, the carbon constituted 14% of the total dust (450 μg/m^3^). The total carbon (5%) was classified as elemental carbon. The online BC measurements (AE51) showed lower BC concentrations (2 μg/m^3^ compared to 23 μg/m^3^). The two methods are based on very different principles, and the online concentrations measured by the AE51 should hence only be used for the purpose of studying relative variations over time, not absolute numbers.

The measured concentrations were in the same range as previously reported in an earlier study at the same site and collecting area 1 year earlier (*c.f.* TD 5.1 mg/m^3^, RD 0.48 mg/m^3^, and PN 24 000 particles/cm^3^).^4^ While the mass was dominated by particles sized >1 μm, most of the formed particles (by number) was smaller than 100 nm (77%), and nearly all particles was smaller than 1 μm. Since these small particles can have a long residence time in the atmosphere and are small enough to be inhaled,^28^ they risk being diffusively dispersed to the environment and cause harm. Such effects on zooplankton and fish are presented in Sections 3.2–3.4. Even though the particle concentrations varied during the measurements, the size distributions were very stable (i.e., count median particle diameter and standard deviation of the distribution) (Figure 2).

The results are in line with recent literature findings on WEEE particle aerosols generated from dismantling, shredding, and mechanical grinding of, e.g., computer monitors, fluorescent lamps, and printed circuit boards. Large variations in particle size distributions, depending on the type of WEEE material, are reported with some particles being in the respirable range (<2.5 μm). Certain stages of the dismantling process, such as shredding and grinding, results in increased particle concentrations with sizes in the respirable range.^4^

Hence, it can be concluded that the recycling of electrical and electronic equipment results in the formation of both nano- and submicrometer-sized particle aerosols that, due to their physical particle characteristics, may be inhalable and can potentially reach the deep parts of the lung. Moreover, due to the open waste recycling facility and the long residence time of the particle aerosols, they are also likely to be dispersed into the environment.

### 3.2 WEEE Particle Characteristics: Shape and Elemental Composition

**Figure 4:**
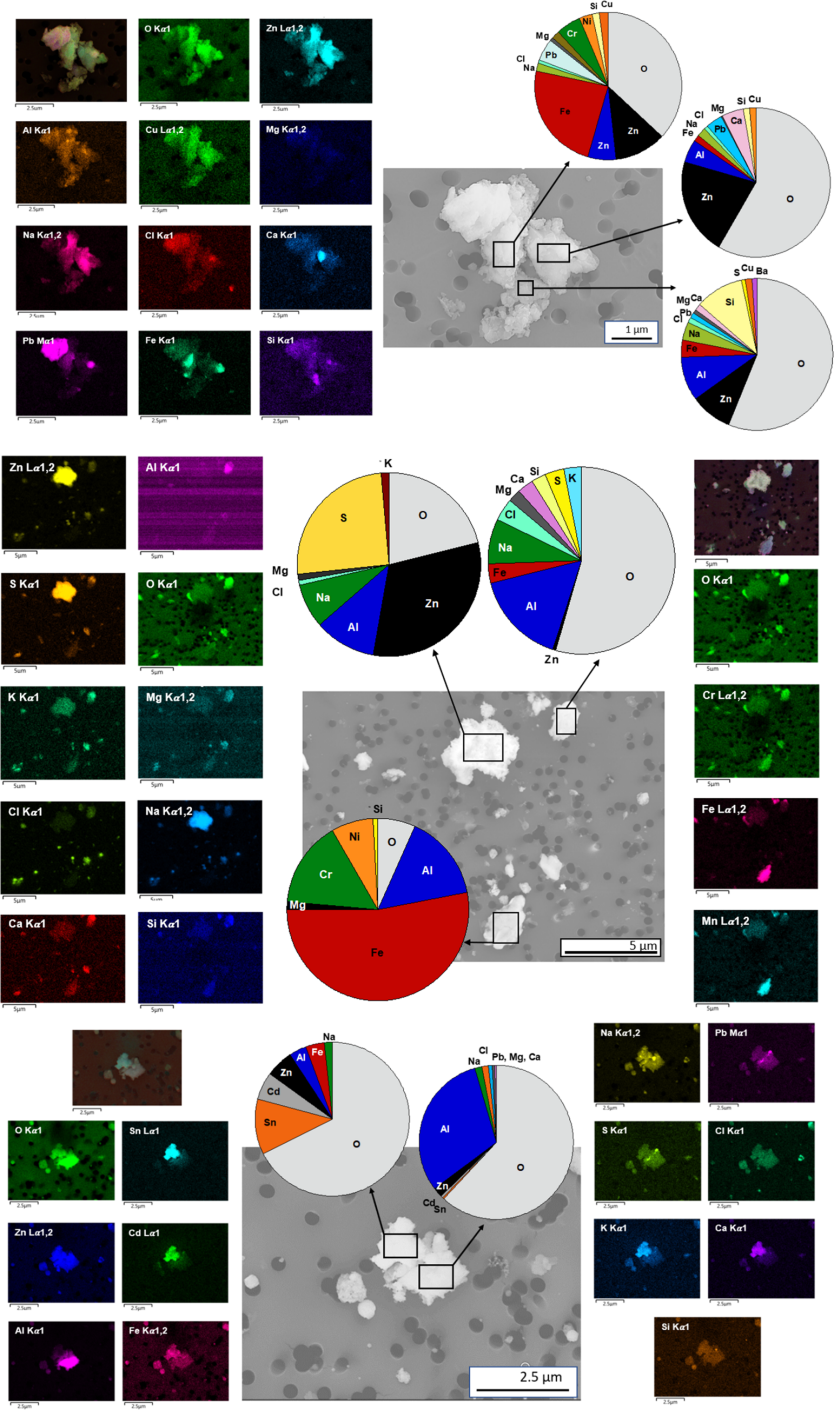
Typical morphology and relative mass composition (area analysis of eight different agglomerates) of collected WEEE aerosol particles/aggregates determined by means of SEM/EDS.

The collected particles revealed a large variety in terms of shape, size, composition, and extent of aggregation. Typical collected WEEE aerosol particles and aggregates possible to observe and analyze by means of SEM/EDS are illustrated in Figure 4 together with corresponding elemental maps. In addition to oxygen (O), the particle agglomerates were composed of inorganic constituents including sodium (Na), calcium (Ca), potassium (K), chlorine (Cl), and sulfur (S) as well as metal(oids) such as silicon (Si), Fe, Al, Zn, Cr, and to a lesser extent Cu, Pb, magnesium (Mg), Ni, Ba, tin (Sn), and Cd. Since the filters used for collecting the particles made of polycarbonate contains O, the levels of O may be overestimated.

The figure reveals the compositional complexity of the collected particles, reflected by the varying composition of the electronic waste. The observed elements are in line with previous literature findings.^2,29^ Some of the observed metals, including Cd, Cr, Pb, and Sn, were present in relatively small amounts, although they have known hazardous properties both from a health and environmental perspective.^30^ These metals can for example originate from electronic switches (Cd), solder joints and wire insulation (Cd), metal housing (Cr), solders on PC boards (Pb and Sn), and cathode ray tubes (Pb and Ni). Other metals such as Cu and Zn are essential metals but can, dependent on concentration and chemical form, induce adverse effects on living organisms. Zinc (as Zn sulfide) is for example used as a luminescent pigment in cathode ray tubes. Copper is used in wires and cables, PC-boards, relays, switches, electromagnetic motors and Pb-free solders due to its superior conductivity of heat and electricity.

The inorganic metalloids and elements such as Ca, Si, and Cl are the main constituents of printed circuit boards, which consist of woven glass fiber sheets hardened (e.g., Ca, Al, and Si oxides) with flame retarded epoxy resins (e.g., Br and Cl) and traces of Cu.

**Figure 5:**
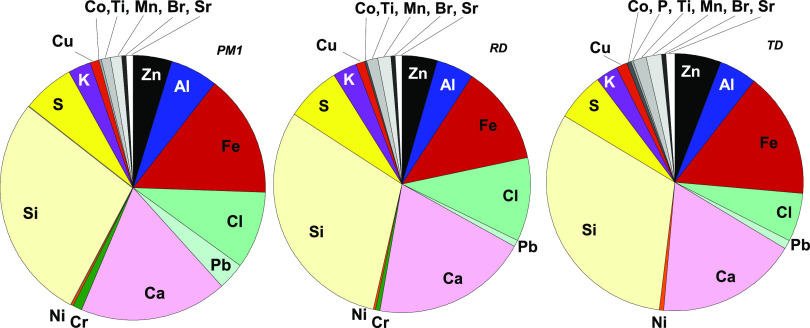
Relative chemical mass composition (except C and O) of collected WEEE aerosols fractioned into PM1 (<1 μm), RD (respirable dust <2.5 μm), and TD (total dust), based on PIXE and ICP-MS analyses.

Elemental compositional analyses of the complete assembly of collected particle mass, separated into the fractions of TD (total dust, very approximately corresponding to particles of diameters <30 μm), RD (respirable dust, <4 μm), and PM1 (<1 μm), were conducted by means of PIXE and ICP-MS. The relative mass fractions of the observed elements are presented in Figure 5. Since only elements with a higher atomic number than 12 can be determined, the results do not include carbon (C), O, or Na, elements observed by means of EDS.

No evident differences in composition between the particle fractions could be observed. This observation, as well as the overall chemical content of the particles, are in line with the measurements made 1 year earlier at the same site.^4^ In addition to the elements observed by means of EDS (Figure 4), the PIXE analyses revealed the presence of small amounts of cobalt (Co), phosphorus (P), titanium (Ti), manganese (Mn), bromium (Br), and strontium (Sr) as well as traces of vanadium (V), gallium (Ga), germanium (Ge), arsenic (As), selenium (Se), rubidium (Rb), yttrium (Y), zirconium (Zr), niobium (Nb), molybdenum (Mo), Cd, Sn, antimony (Sb), Ba, tantalum (Ta), and tungsten (W).

The existence of these elements in WEEE particles is not surprising as they are all present and have various function in electronic components, e.g., Br in flame retardants in plastics and foams, Sr in cathode X-ray tube windows, Se in photocopying machines and photocells, Y in cathode ray tubes, and As and Ga (as gallium arsenide) in semiconductors in integrated circuits, infrared light emitting diodes, laser diodes, and solar cells.

The inorganic elements analyzed by PIXE and ICP-MS cover ∼40 to 50% of the gravimetric mass. The fraction of organic carbon corresponded to 13% of the gravimetric mass (elemental carbon, EC 1%). Assuming that the elements analyzed are in the form of the most commonly occurring oxides (a 1:1 ratio of C and O was assumed), the mass recovery was ∼90%.

In all, it can be concluded that the WEEE particle aerosols formed show a large variety in particle size, shape, and chemical composition comprising both nonessential and essential metals and metalloids. In the complete assembly of the collected particles, the most abundant elements were Si, Ca, Fe, Cl, S, Zn, and Al in decreasing order followed by K, Cu, Ti, Mn, Br, Sr, Ni, Cr, and traces of Co, V, Ga, Ge, As, Se, Rb, Y, Zr, Nb, Mo, Cd, Sn, Sb, Ba, Ta, and W. Some of the observed elements have known environmentally hazardous properties at sufficient concentrations and chemical forms.

### 3.3 WEEE Particle Characteristics: Surface Composition and Oxidation State

**Figure 6:**
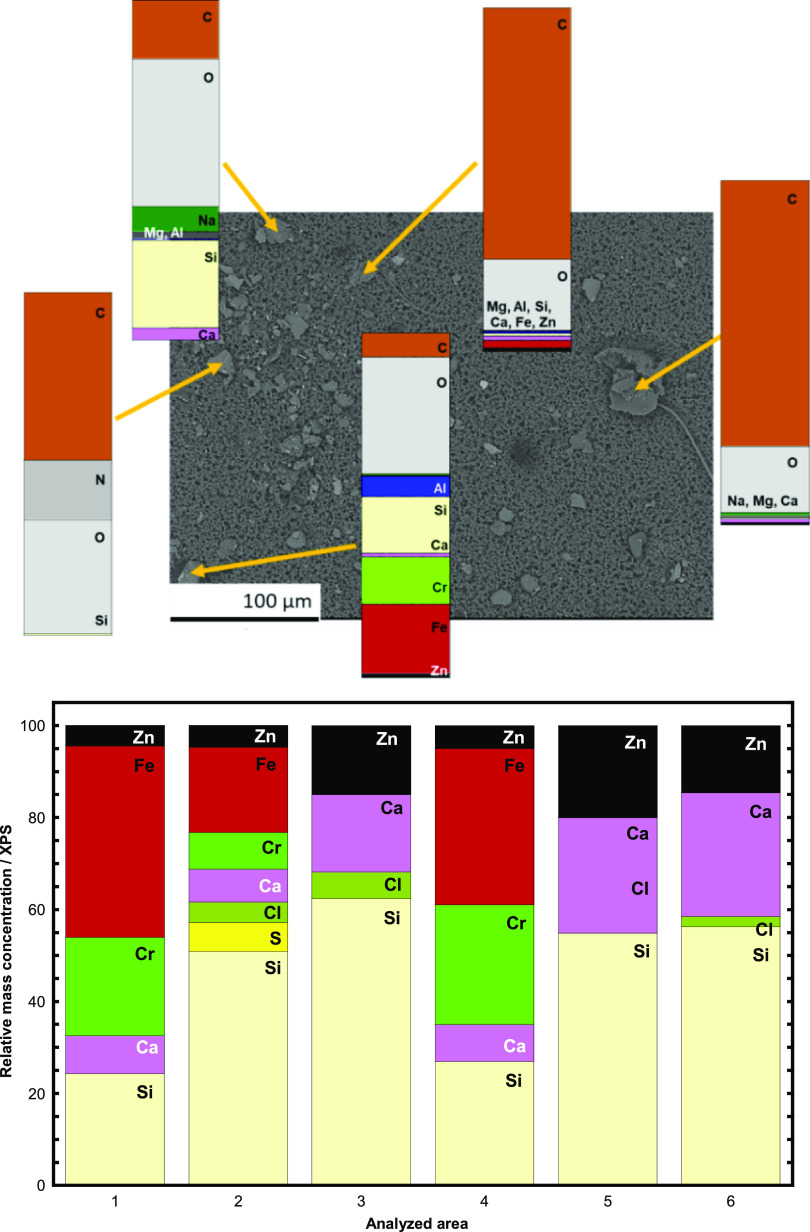
Relative elemental composition by means of EDS (top) of separate particles and average relative mass composition (oxygen and carbon excluded) by means of XPS (bottom) on an assembly of particles (RD) collected on the same filter.

XPS measurements were conducted on an assembly of collected particles from one of the filters corresponding to respirable dust (Figure 6). The same filter was also analyzed by means of EDS showing some particles containing mainly C and O with small amounts of Mg, Al, Si, Ca, Fe, and Zn, and other particles composed of C, O, Na, and Si with small amounts of Mg, Al, and Si, and particles mainly composed of O, Si, Fe, and Cr, with small amounts of Al, Ca, and Zn. Similar compositional observations were observed using EDS and XPS. From the observed binding energies of the XPS findings, all metals and metalloids were present in their oxidized state, e.g., as Zn(II), Fe (II,III), Ca(II), Al(III), Si(IV), and Cr(III).

The chemical speciation of Zn, Cu, Cr, and Fe in the collected particles was further investigated by means of XANES on TD filters and the impactor stages, see Materials and Methods. Representative XANES spectra are given in the Supporting Information (Figure S1).

For Fe, the energy of the K-edge (defined at the half-height of the main peak) can be used to estimate the oxidation state of Fe. The analysis indicated an oxidation state between (II) and (III), i.e., (+2.7) with spectra similar to Fe_3_O_4_ and Fe_2_O_3_ for all samples analyzed. The spectra for TD and the particles from the impactor stages, corresponding to particles size fractions of 0.20–4.5 μm, were close to identical and all with the same oxidation state, indicating the same chemical form for the different particle sizes. These results are in line with previous findings of particles collected at the same occupational setting.^4^ The particles extracted from the collecting filters using methanol (for the ecotoxicity studies) showed nearly identical Fe-XANES spectra.

Since the concentrations of Cr were low and the element not present in all particles/agglomerates (see Figures 4 and 6), not all samples could be analyzed. Nevertheless, the impactor stage particles, which contained Cr, corresponded to particles sized ∼2 μm with the oxidation state (III). The acquired spectra resembled the reference spectra of NiCr_2_O_4_, which implies the presence of Cr in a mixed oxide (a spinel) such as, e.g., NiCr_2_O_4_. These results are in line with the SEM/EDS findings showing particles containing Fe, Cr, and Ni (Figure 4), which indicate an origin from stainless steel in which Cr is present as Cr(III) in the passive surface oxide. No indication of the more toxic form of Cr in the oxidation state (IV) (10–100 times more toxic than Cr(III))^31^ was observed. The methanol extracted particles (<1 μm) showed the same chemical form of Cr as the ∼2 μm particles, i.e., oxidation state (III). For the particles collected on the impactor stage corresponding to a size of ∼300 μm, a slight difference in the features of the XANES spectra were observed, indicative of a difference in chemical form but with the same oxidation state, i.e., (III).

Cu showed more variation in chemical form between the investigated particle size fractions. The main difference was observed for the particles sized 1.5–2.7 μm, 2.7–4.5 μm, and TD (typically dominated by the mass of even larger particles). The major differences fit very well with an increased fraction of Cu in its metallic state (oxidation state (0)), increasing from 10 to ∼50% for both the ∼2 μm particles and TD. Apart from metallic Cu (oxidation state (0)) in the largest particles, the dominating oxidation state for the smaller particles was (II), indicative of Cu metal or Cu alloy particles with oxidized surfaces. No effect of the methanol filter extraction was observed in terms of chemical form for the particles sized less than <2.7 μm.

The largest variation in the XANES spectra, over particle size, was observed for Zn. Zn was mainly observed in the oxidation state (II) for particles sized <4.5 μm, but the shape of the XANES spectra varied between the particle size fractions of 100–150 nm, 220–360 nm, and 1.5–2.7 μm, indicative of a difference in chemical form but with the same oxidation state (II). This could be related to the presence of Zn as zinc sulfide as this compound is used as a luminescent pigment in cathode ray tubes, see discussion above. Zinc and sulfur were also identified to a large extent in some particles/aggregates. The spectra of the 2.7–4.5 μm particles were identical to the 1.5–2.7 μm particles. Zn in its metallic form, oxidation state (0), was observed in the TD particles. The spectra matched with the reference spectra of brass (up to 30%), though the origin can also be other Zn metal containing materials. No changes in chemical form was observed for the methanol-extracted particles. The spectra were similar to those of the TD particles, but not when compared with the particle (PM1) dispersions prepared for the ecotoxicity testing, which was the case for Cu, Cr, and Fe. This could possibly be explained by the loss of soluble Zn being potentially adsorbed to either the filter surface or the glass vessel walls during the extraction procedure and stock-solution preparation.

Overall, the observed metals and metalloids of the WEEE particles were mainly present in their oxidized state, e.g., as Zn(II), Cu(II), Fe (II,III), Ca(II), Al(III), and Cr(III). The largest particles also revealed Cu and Zn in their metallic forms, which reflect a metal core with oxidized surfaces. The XANES spectra of the pristine particles sized ∼1 to 2 μm of Cu, Cr, and Fe coincided with observations of the particles after methanol extraction and in the stock solutions used for the ecotoxicity testing. These results indicate that the preparation steps did not induce any substantial changes in the chemical form of either Cr, Fe, or Cu. The minor difference observed for Zn could possibly be explained by the loss of water-soluble Zn during the extraction of particles and particle dispersion preparation.

### 3.4 WEEE Particles: Ecotoxicological Potency

#### 3.4.1 Metal Composition of Extracted Particles in Stock Solution

**Figure 7:**
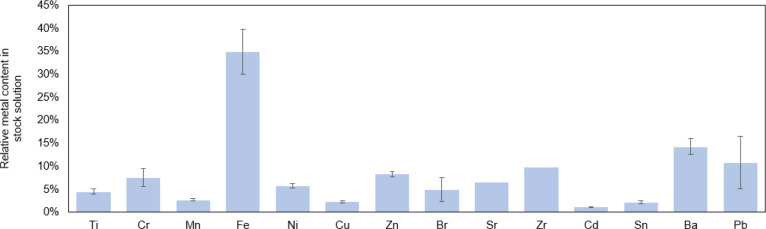
Relative mass content of main metals in the stock solution of the extracted WEEE particles sized <1 μm for the ecotoxicity measurements determined by means of PIXE.

The relative metal content in the extracted WEEE particles dispersed into stock solutions (see Materials and Methods) are presented in Figure 7, excluding the presence of Al, Si, and Ca. Similar to the findings illustrated above, the assembly of the particles contained several different metals.

**Figure 8:**
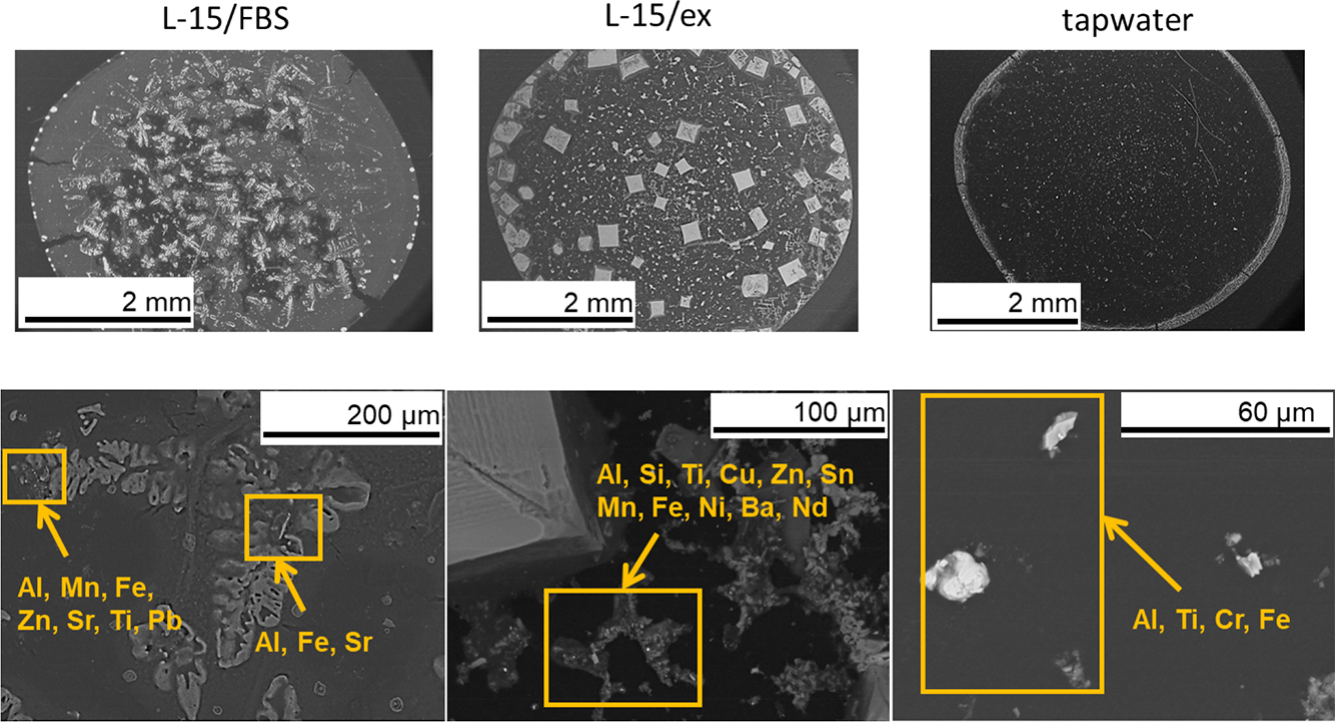
Metals in the extracted WEEE particles sized <1 μm for the ecotoxicity measurements observed by means of EDS in different particles/agglomerates in the different exposure media for the rainbow trout cells (L-15/FBS and L-15/) and *D. magna* (tap water) studies. The dried crystals in L-15/FBS and L-15/ex reflect the solution components, see Materials and Methods).

SEM/EDS analysis of the extracted particles in the different test solutions used for the Rainbow trout gill cells and *D. magna* studies, respectively (see below), showed, as illustrated above, the same elements to be linked to different particles (Figure 8).

#### 3.4.2 Cytotoxicity

**Figure 9:**
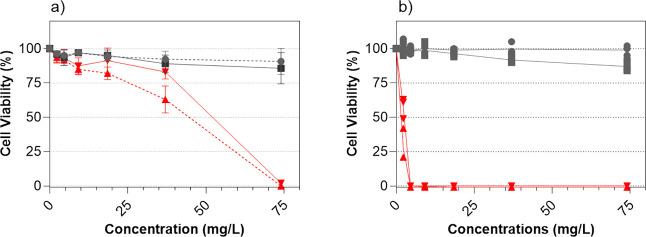
Cell viability of RTgill-W1 cells after a 48 h exposure to increasing concentrations of WEEE particles (2.3–74 mg/L) in (a) L-15/FBS and (b) L-15/ex. Cytotoxicity was assessed compared to CuSO_4_ by means of Alamar Blue and CFDA-AM. Cells incubated with the medium served as negative control and were used in normalization. The results are presented as mean percentages of at least three replicates, and the error bars represent standard errors of the means (SEM) of at least three independent experiments.

In vitro exposure of the extracted WEEE particles in different concentrations (2.3–74 mg/L) to the rainbow trout gill cells resulted in a slightly (though nonsignificant) reduced cell viability only for the highest particle concentration (74 mg/L), but no inherent difference in toxicity was observed between the exposure media (L-15/FBS and L-15/ex) (Figure 9). No ecotoxic effects were observed in any of the tested media for the other particle concentrations tested (2.3, 4.6.9.2, 18.5, 37, and 74 mg/L). The positive control, CuSO_4_, showed higher toxicity in the L-15/ex medium compared to the L-15/FBS medium, with concentrations as low as 12.5 mg/L eliciting a toxic response. Cell exposures in the L-15/ex medium have previously been shown to be more sensitive to chemicals.^27^ This implies that since no pronounced response was observed neither in L-15/FBS nor in the L-15/ex medium, the collected WEEE particles were not toxic at the lower concentrations investigated.

#### 3.4.3 Oxidative Stress

Oxidative stress in RTgill-W1 cells was measured by generation of reactive oxygen intermediates produced on exposure to WEEE particles in different cell media. ROS generation measured after immediate exposure (for a period of 18 h) showed a higher extent of residual ROS and increasingly positive slopes with increasing WEEE particle concentrations.

**Figure 10:**
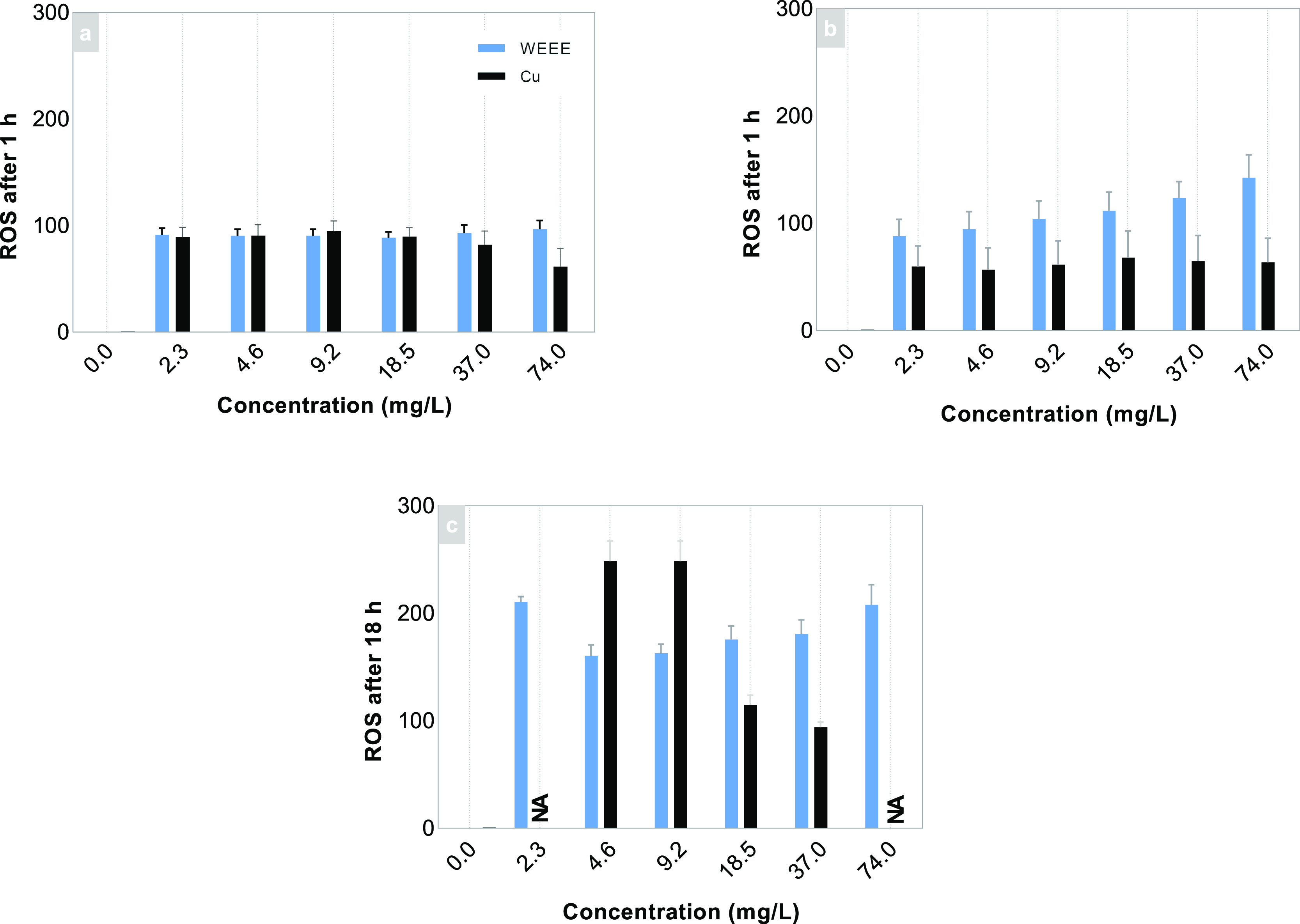
ROS generated over 1 h post 48 h exposure of WEEE particles to RTgill-W1 cells in (a) L-15/FBS medium and (b) L-15/ex media. (c) Relative ROS generated over a 18 h exposure of the WEEE particles in L-15/ex compared with a control (CuSO_4_). Results are presented as average slopes of ROS curves and standard deviation of the means (SEM) of at least three independent experiments.

However, the cytotoxic CuSO_4_ revealed a negative concentration-slope relationship. Cytotoxic effects of CuSO_4_ were evident after the slopes peak at a concentration of 12.5 mg/L (Figure 10) and decreased with increasing concentration, indicative of increased cell damage.

After 48 h of exposure, residual ROS measured over 1 h showed an increased formation with increasing WEEE particle concentration. However, small differences in the extent of residual ROS in L-15/FBS and L-15/ex were observed after 48 h with the highest concentration, 74 mg/L, showing a 2-fold slope difference (Figure 10b). The observed differences between the two exposures in the different cell media are unlikely linked to toxic effects.

In all, the cell exposure to the WEEE particles only showed some (nonsignificant) cytotoxic effects toward *RTgill-W1*cells for the highest particle concentration investigated (74 mg/L), though the exposure resulted in ROS formation that may induce adverse effects.

#### 3.4.4 *D. magna*Acute Toxicity Test

**Figure 11:**
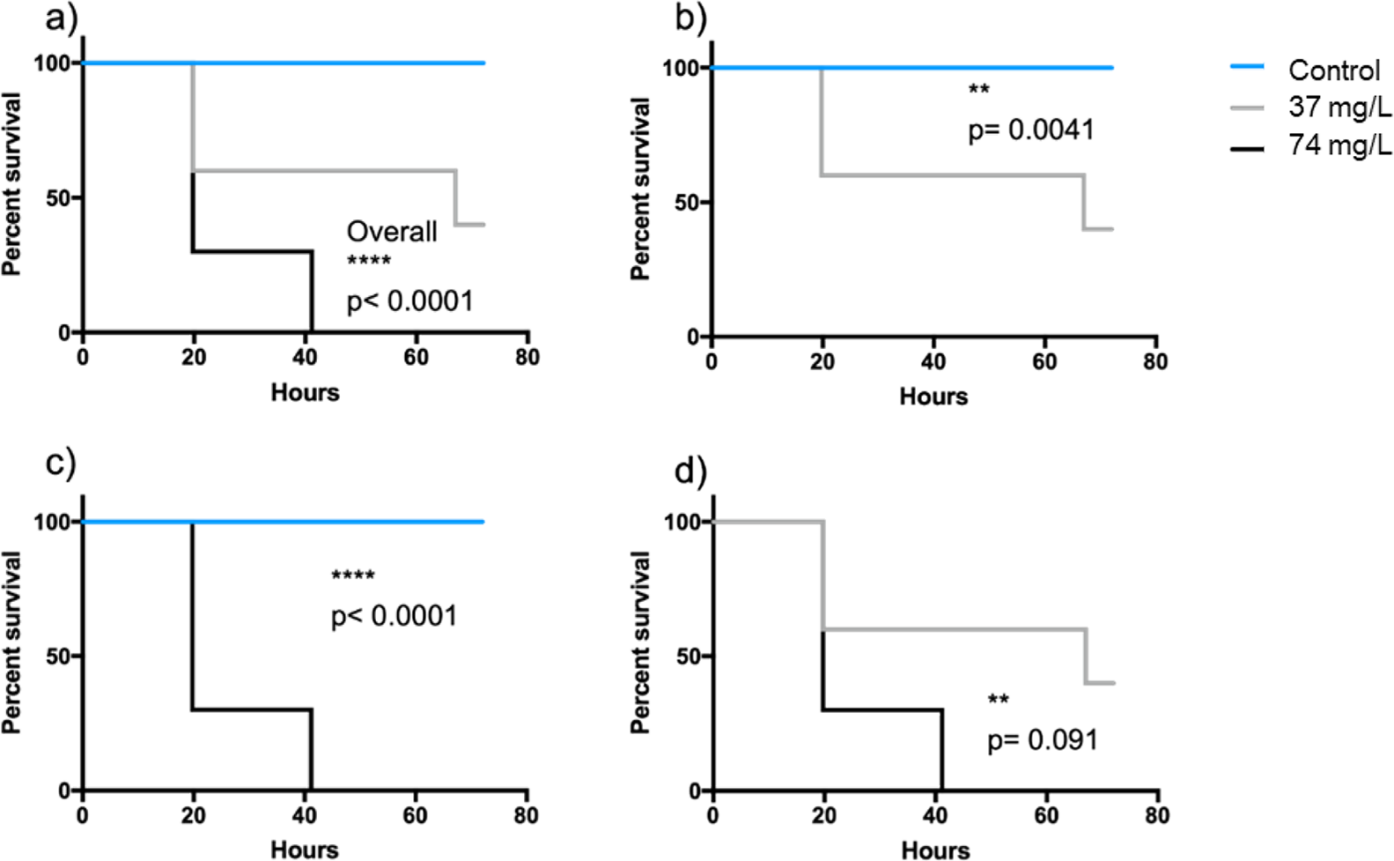
Kaplan–Meier survival curves from a 72 h acute toxicity test using *D. magna*. (a) All treatments, (b) pairwise comparison between control and 37 mg/L, (c) pairwise comparison between control and 74 mg/L, and (d) pairwise comparison between 37 and 74 mg/L. *p* denotes the *p*-value from the statistical test, and stars denote statistically significant differences. Blue lines = control, gray lines = 37 mg/L, and black lines = 74 mg/L.

The toxicity test on the zooplankter *D. magna* revealed an overall significant difference in survival rate for both particle concentrations (37 and 74 mg/L) (χ^2^_(2_) = 22.99, *p* < 0.0001) (Figure 11a). Pairwise comparisons further revealed that there were significant differences between all tested treatments. At the highest concentration, no individual *Daphnia* survived even 40 h of exposure, whereas at the lower concentration, more than 50% of the individuals were alive after an 80 h exposure (Figure 11 b–d).

In all, the zooplankton bioassay shows that the WEEE particles were indeed toxic toward *D. magna* in a concentration-dependent way. This is in accordance with a previous study showing acute toxic responses to several of the metals we identified as components in the WEEE particles.^32^ The underlying mechanisms behind the observed toxicity and potential effects arising from cocktail effects due to the diverse mixture of metal NPs in the WEEE dispersion need to be further explored.

## 4 Concluding Remarks

Overall, our studies highlight the importance of systematic investigations of the physicochemical characteristics of WEEE particles and their toxic potency toward different aquatic recipients. Such information is crucial for the development of appropriate regulations and guidelines for safe handling and disposal of electronic waste. It is also essential from an environmental fate perspective and to ensure sustainable production patterns, thereby achieving the global sustainability goals set by the United Nations, such as Sustainable Cities and Communities (#11) and Responsible consumption and Production (#12).

Our study further shows that the recycling treatment of electric and electronic waste results in the formation of WEEE particles, which to a large extent are of inhalable sizes (<1 μm). The particles formed have a complex chemistry and are composed of a multitude of organic and inorganic components (metals and metalloids). Depending on composition and dose, these particles can if dispersed into the environment induce toxic effects on aquatic organisms, as shown for the zooplankton *D. magna*, whereas no significant toxic effects were recorded in a rainbow trout cell line. The underlying mechanisms remain to be further explored.

Future studies should assess the most important environmental dispersion pathway for WEEE particles.

## Supporting Information Available

XANES spectra of Fe, Cu, Cr, and Zn edges for the WEEE particles (PDF)
