# Supplementary material for: Characterization and Toxic Potency of Airborne Particles Formed upon Waste from Electrical and Electronic Equipment Waste Recycling: A Case Study
Source: ACS Environ Au. 2023 Nov 3;3(6):370–82. doi: 10.1021/acsenvironau.3c00034 (PMC10655593; doi:10.1021/acsenvironau.3c00034)
Supplement: Supplementary file 1 — vg3c00034_si_001.pdf [file vg3c00034_si_001.pdf]

Supporting information

Characterization and toxic potency of airborne particles formed upon WEEE waste recycling – a case study

Inger Odnevall[1,2,3]*, Marianne Brookman-Amissah[5], Franca Stabilé[6], Mikael T. Ekvall[6,9], Gunilla Herting[1], Marie Bermeo Vargas[7,9], Maria E. Messing [7,9], Joachim Sturve[5], Lars-Anders Hansson[6], Christina Isaxon[4,9], Jenny Rissler[4,8,9]*

[1]*KTH Royal Institute of Technology, Department of Chemistry, Division of Surface and Corrosion Science, SE-100 44 Stockholm, Sweden*

[2]*AIMES–Center for the Advancement of Integrated Medical and Engineering Sciences at Karolinska Institute and KTH Royal Institute of Technology, SE-100 44 Stockholm, Sweden*

[3]*Karolinska Institute, Department of Neuroscience, SE-171 77 Stockholm, Sweden*

[4]*Ergonomics and Aerosol Technology, Lund University, 22100 Lund, Sweden*

[5]*University of Gothenburg, Department of Biological and Environmental Sciences, Gothenburg, Sweden*

[6]*Department of Biology, Aquatic Ecology, Lund University, Lund, Sweden*

[7]*Solid State Physics, Lund University, Box 118, 221 00 Lund, Sweden*

[8]Bioeconomy and Health, RISE Research Institutes of Sweden, 22370 Lund, Sweden

[9]NanoLund, Lund University, SE22100 Lund, Sweden

**Correspondence: jenny.rissler@ri.se (J.R.); ingero@kth.se (I.O.)*

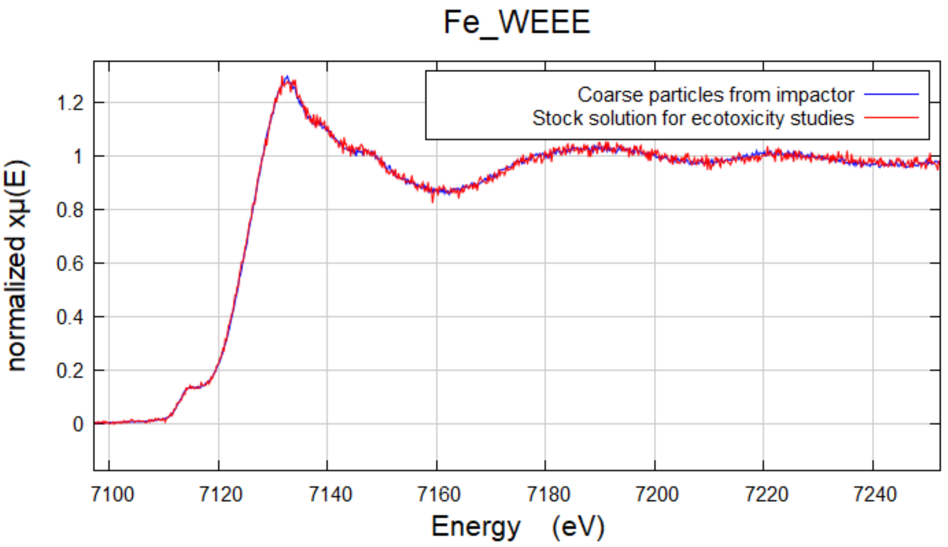

Figure S1. XANES spectra at the Fe K-edge (7112 eV) for untreated aerosol particles (from impactor stage corresponding to 1.5-2.7 µm particles), and for stock solution prepared for the ecotoxicity studies. The similarity in XANES spectra indicate that the preparation steps did neither alter the oxidation state nor the chemical form of Fe.

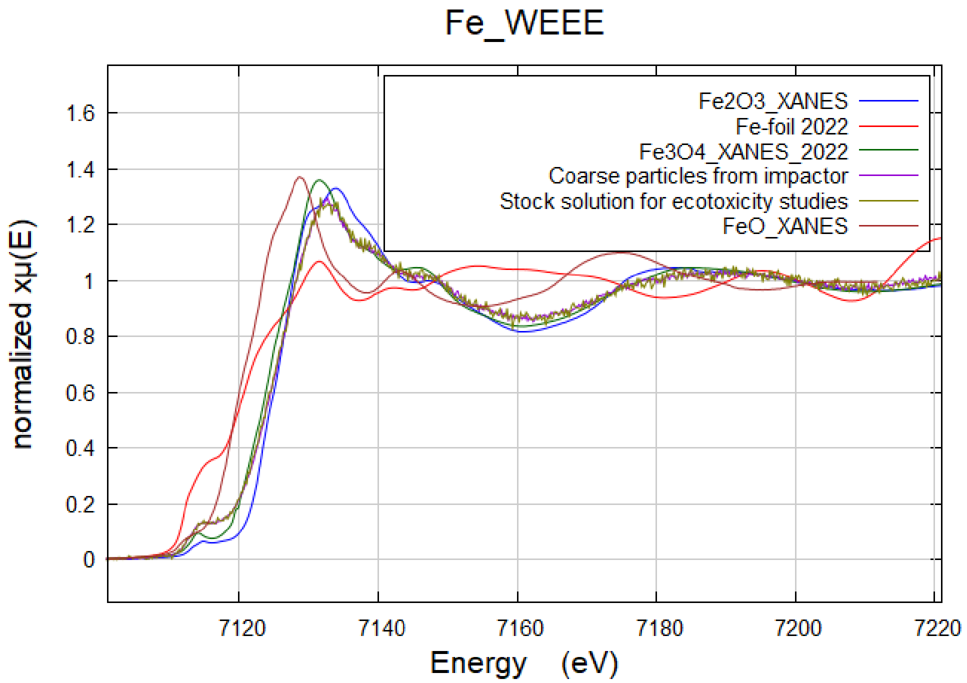

Figure S2. Same as in A1 including a few selected reference spectra for comparison to show the variation in XANES spectra with chemical form.

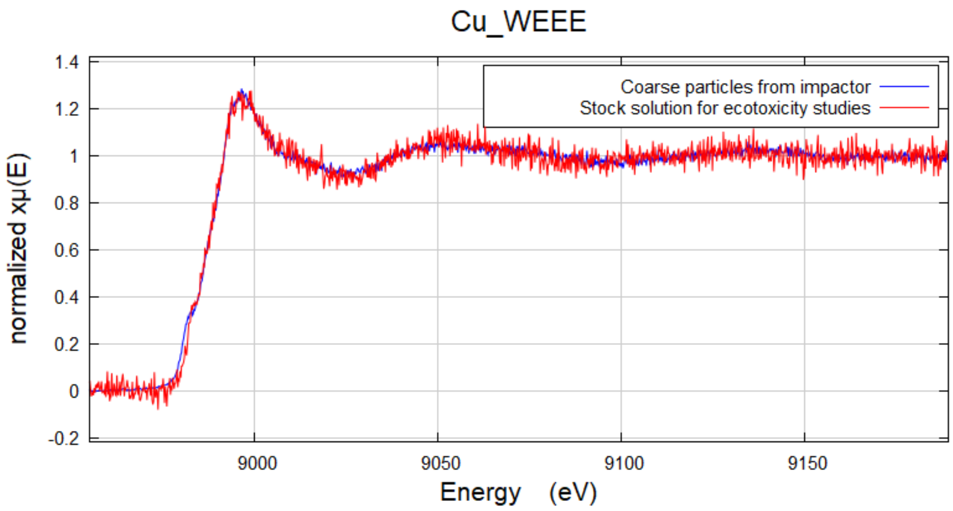

Figure S3. XANES spectra for the Cu K-edge (at 8979 eV) for untreated aerosol particles (from impactor stage corresponding to 1.5-2.7 µm particles), and for stock solution prepared for the ecotoxicity studies. The similarity in XANES spectra indicate that the preparation steps did not alter oxidation state nor the chemical form of Cu.

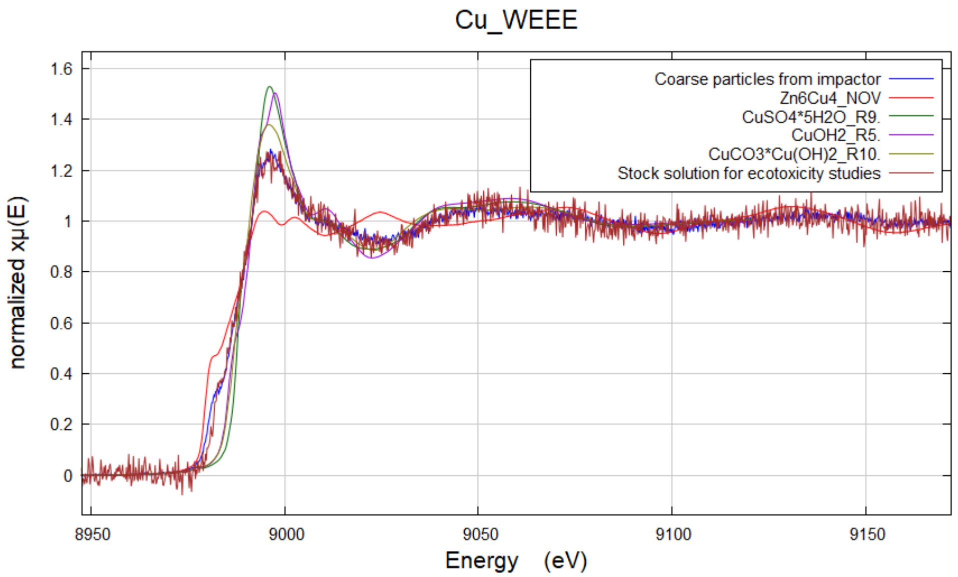

Figure A4. Same as in A3 including a few selected reference spectra for comparison to show the variation in XANES spectra with chemical form.

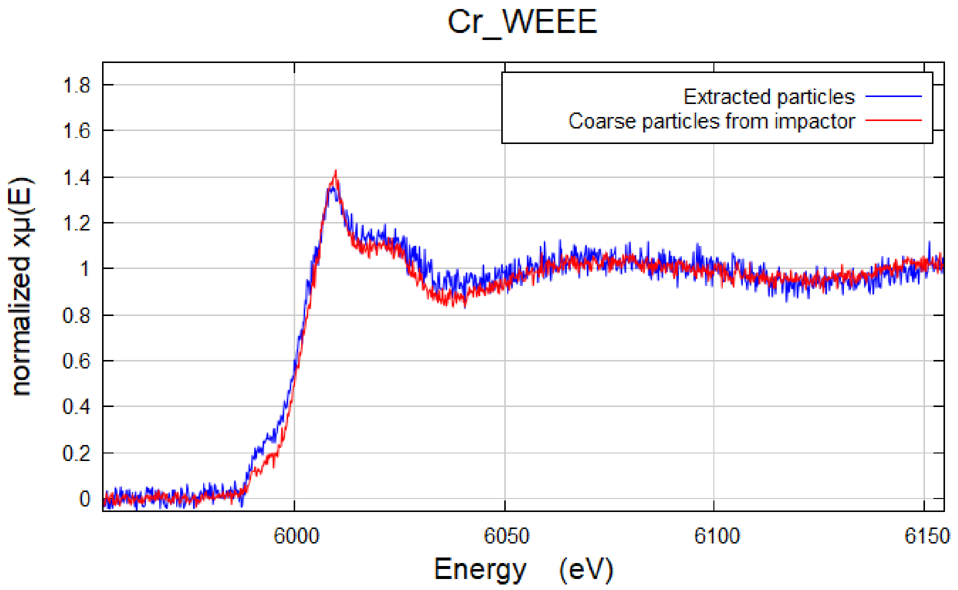

Figure A5. XANES spectra for the Cr K-edge (at 5989 eV) for untreated aerosol particles (from impactor stage corresponding to 1.5-2.7 µm particles), and for extracted particles. No spectra for stock solutions could be retrieved due to low concentrations. Some minor differences in XANES spectra were observed, but in principle no change in oxidation state of Cr was detected.

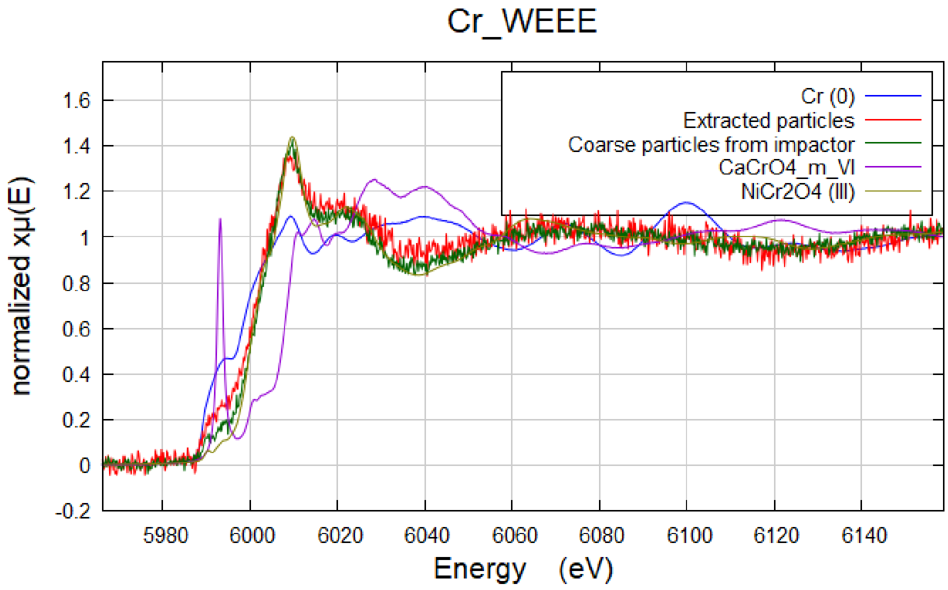

Figure A6. Same as in A5 including a few selected reference spectra to illustrate the typical variation in XANES spectra with chemical form.

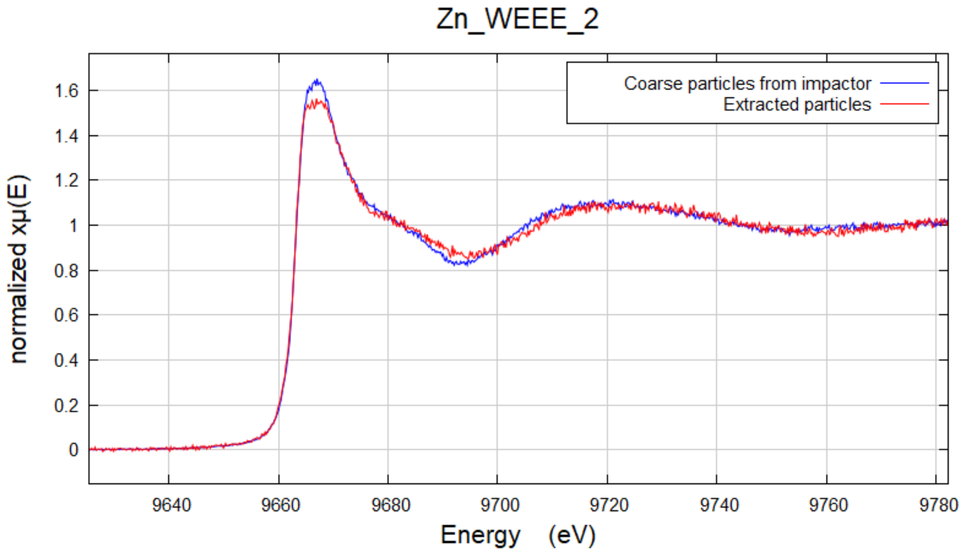

Figure A7. XANES spectra for the Zn K-edge (at 9659 eV) for untreated aerosol particles (from impactor stage corresponding to 1.5-2.7 µm particles), and for stock solution prepared for the ecotoxicity studies. Some minor differences in XANES spectra were observed comparing the Zn spectra for the impactor stage and stock solution.

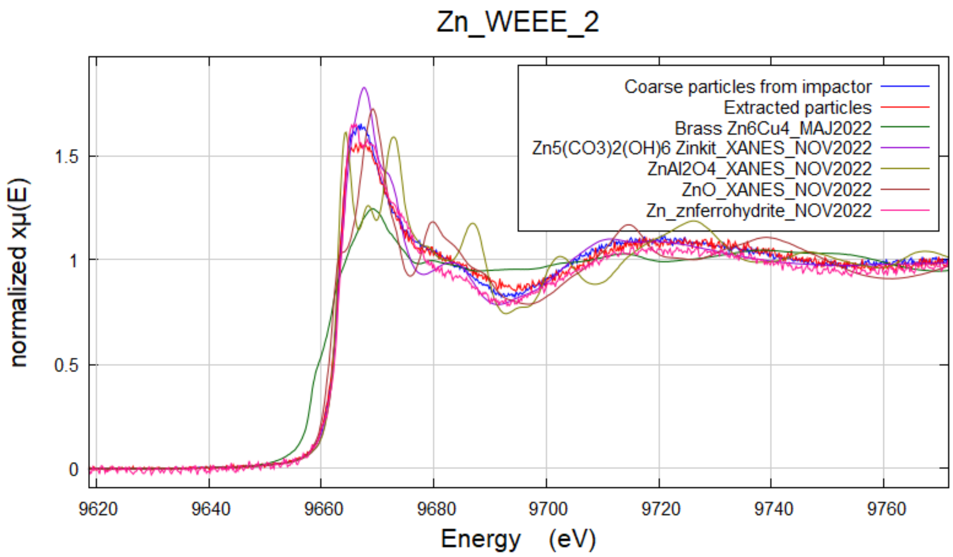

Figure A8. Same as in A7 including a few selected reference spectra to illustrate the typical variation in XANES spectra with chemical form.
